# Unzipping Zipf’s law

**DOI:** 10.1371/journal.pone.0181987

**Published:** 2017-08-09

**Authors:** Sander Lestrade

**Affiliations:** Centre for Language Studies, Radboud University, Nijmegen, The Netherlands; The University of Memphis, UNITED STATES

## Abstract

In spite of decades of theorizing, the origins of Zipf’s law remain elusive. I propose that a Zipfian distribution straightforwardly follows from the interaction of syntax (word classes differing in class size) and semantics (words having to be sufficiently specific to be distinctive and sufficiently general to be reusable). These factors are independently motivated and well-established ingredients of a natural-language system. Using a computational model, it is shown that neither of these ingredients suffices to produce a Zipfian distribution on its own and that the results deviate from the Zipfian ideal only in the same way as natural language itself does.

## Introduction

George Kingsley Zipf (1902-1950) famously observed that the frequency of occurrence of words is neither uniformly nor normally distributed, but inversely related to their frequency rank instead [1]. That is, using a text dependent parameter *C*, the frequency of word *i* corresponds to the division of *C* by the rank position of *i*:

$$\begin{array}{c} frequency_{i}=C/rank_{i} \end{array}$$ (1)

**Fig 1:**
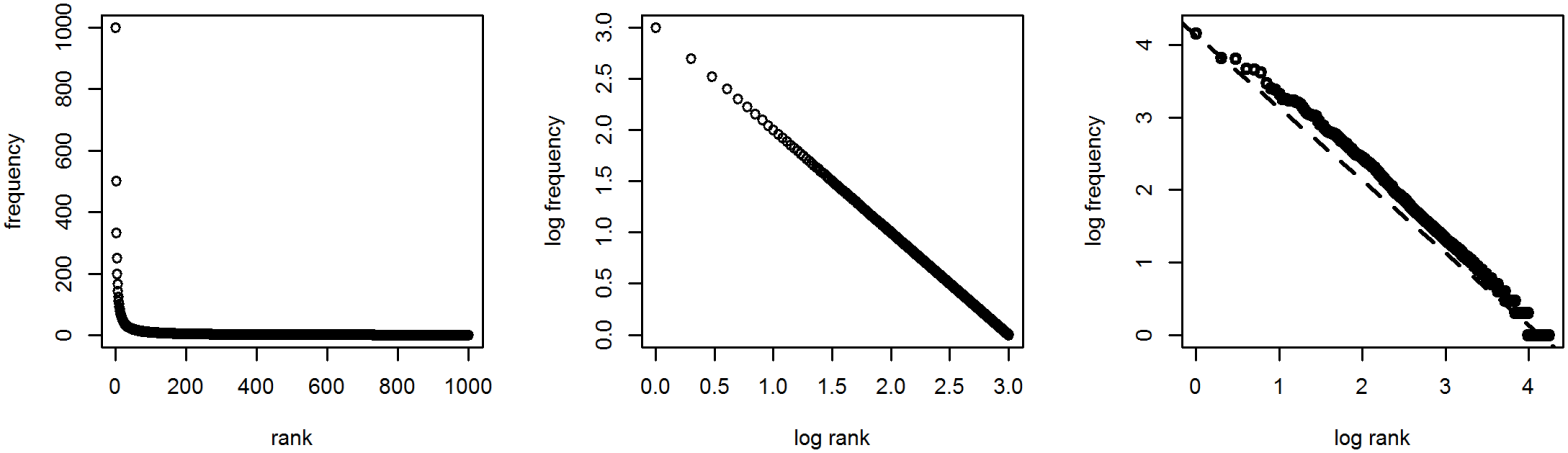
Zipf’s law. A: Predicted frequency by rank. B: Predicted frequency by rank in double-log space. C: Frequency development in Melville’s *Moby Dick*.

The corresponding *Zipfian distribution* is given on the A panel of Fig 1. It is more commonly presented in double-log space (panel B) in which it forms the straight line that is characteristic for power laws [2]. Zipf’s law is a special type of power law, however, namely one in which the slope of this line in a plot with equal axes is –45°; a defining, but often overlooked characteristic. On panel C, a natural-language distribution is shown for comparison (viz. Melville’s *Moby Dick*). As can be seen, natural language seems to behave according to Zipf’s law indeed.

Zipf’s empirical observation of the relation between the frequency of occurrence of a word and its frequency rank probably is “the most well-known statement of quantitative linguistics” [3]. First observed in linguistics, the distribution was soon recognized in other disciplines too. In fact, Zipfian distributions are claimed to be “about as prevalent in social sciences as Gaussian distributions are in the natural sciences […], which implies that Zipf’s Law captures a very fundamental regularity in the universe surrounding human beings” [4]. As could be expected then, there is a vast literature on Zipfian distributions. But as several reviews conclude, in spite of the amount of work on Zipf’s law, no satisfactory account has been given and its origins still remain controversial (cf. [5–7]). For example, Piantadosi notes that “essentially all of the work in language research has focused solely on deriving the law itself in principle; very little work has attempted to assess the underlying assumptions of the hypothesized explanation” [7]. What is crucially needed, Piantadosi argues, is providing evidence for the cognitive validity of the proposal. This paper directly responds to this call to action, proposing a linguistically informed explanation in which the distribution follows from the interaction between syntax and semantics. After briefly explaining the idea, I will show how it qualifies both descriptively *and* in terms of the validity of its ingredients.

## Materials and methods

Zipf’s law follows from the interaction between syntax and semantics, and neither of them is sufficient. As for syntax, language makes use of different word classes to build sentences. Whereas these word classes, or *parts of speech* (*POS*), are used with a comparable overall frequency, they differ hugely in class size. For example, there are only three articles in English (*the, a, an*) but probably more than 10,000 nouns. Therefore, an article will be more frequently used than the average noun. Within word classes, some words apply more often than others because of their meaning. As *thing* is a more general noun than *submarine* (the set of objects the former can refer to in fact includes the referent set of the latter), it can be expected to be used more often. Words shouldn’t be too general, however, as this would lead to ambiguity. In order to become frequent (within a word class), a word should be specific enough to single out its referent in context and general enough to be applied to different referents.

For both of these observations there is independent and well-established evidence. In the next sections, it will first be shown how syntax and semantics can be modeled and that neither of them is sufficient to explain Zipf’s law on its own. Next, I will show their interaction does produce a near-Zipfian distribution, only deviating from the ideal in the way natural language does.

### Syntax

With the present availability of large language corpora that are annotated for POS, it is easy to show that word classes vary in size by orders of magnitude. For present purposes, it is irrelevant which word classes are used how frequently exactly; the important point is that all natural languages make use of different word classes, and that the number of items in these classes is extremely different indeed. Table 1 gives an overview of the major POS classes that are recognized in the *Corpus of Spoken Dutch* (*CGN*, 8.6M words; [8]), the Brown corpus (1.1M words; [9]), and the Hungarian National Corpus (*HNC*, 187M words of which only the Hungarian-press subcorpus is used; [10]; all data used in this paper are open-access available through third parties; cf. Section S2 File for repositories.) As can be seen, in each language the difference in overall class frequency is negligible in comparison with the difference in class size.

**Table 1:** Overall frequency per 100 words and size of main POS classes in Dutch (CGN), English (Brown), and Hungarian (HNC). Sorted by average expected word frequency. For Hungarian, only the Hungarian-press subcorpus is used. POS abbreviations: ART article, PRO pronoun, CON connective, P adposition, INT interjection, ADV adverbial, NUM numeral, V verb, A adjective, N noun. [8–10].

Dutch (8.6M)			English (1.1M)			Hungarian (71M)		
pos	freq	members	pos	freq	members	pos	freq	members
ART	6.02	5	ART	1.43	6	DET	12.59	2
PRO	17.79	86	CON	2.17	66	CON	7.14	71
CON	6.66	33	DET	2.04	72	PRO	4.84	216
P	9.09	72	P	4.16	164	P	1.82	289
INT	9.01	774	PRO	2.77	146	NUM	2.83	3,937
ADV	11.99	1,121	ADV	7.50	2,225	Adv	7.67	11,896
NUM	1.40	466	V	23.72	12,130	V	10.98	34,679
V	17.48	24,371	A	11.45	8,435	A	11.14	58,765
A	6.45	15,681	NUM	2.36	1,747	N	29.43	193,252
N	13.09	91,762	N	37.60	34,017			

**Fig 2:**
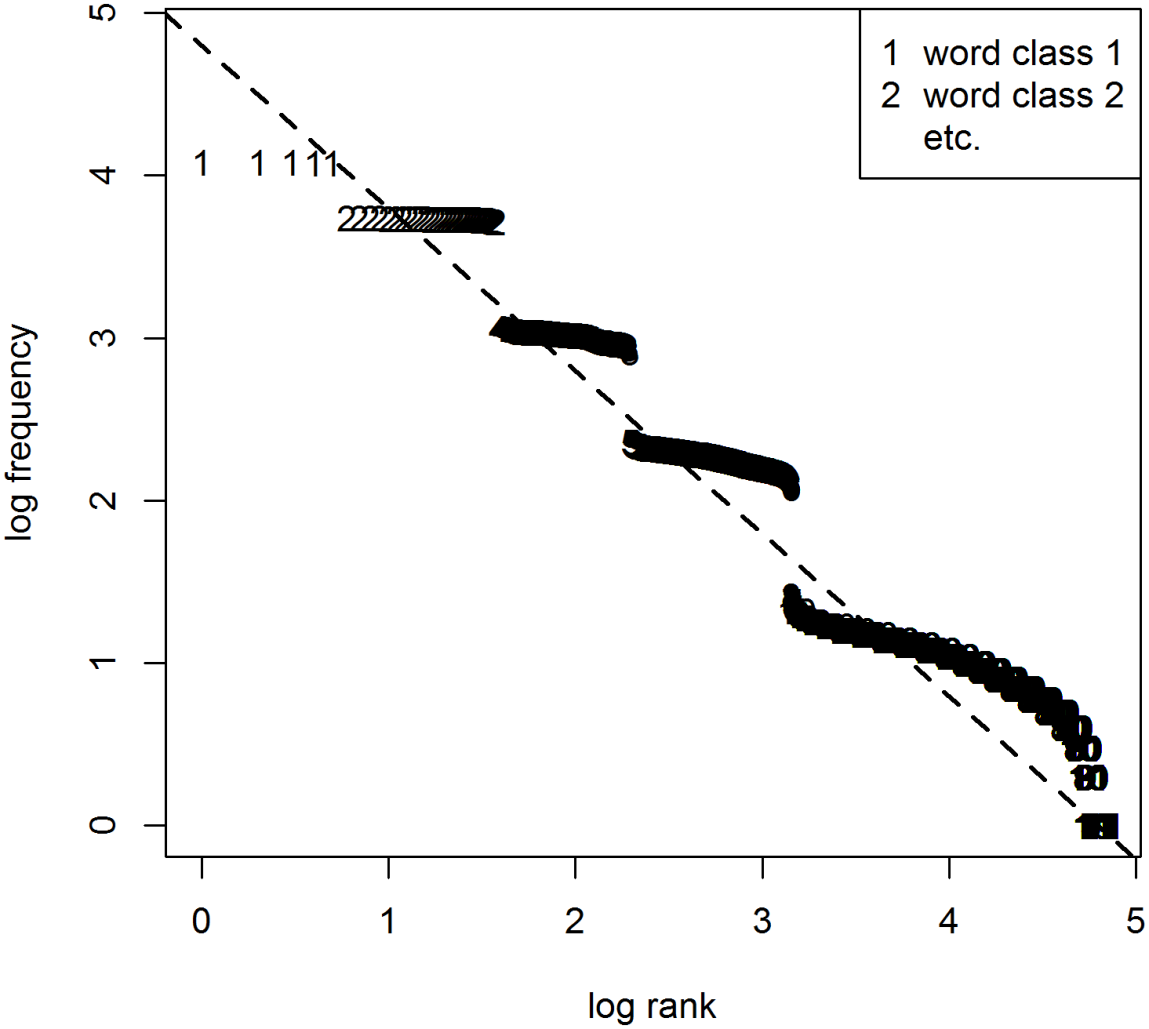
Attempt to generate a Zipfian distribution with syntax only. To generate these results, the class frequencies and class sizes reported for Dutch in Table 1 are used. Numbers correspond to word classes when ordered by expected frequency.

If word class was the only factor at play, a Zipfian distribution would follow from sampling a number of items from each class that is proportional to the overall class frequency. For example for Dutch, to simulate a corpus of 100 words, we should randomly draw (with replacement) six articles from a set of five, 18 pronouns from a set of 86, etc. (cf. Table 1). Fig 2 shows the results of this procedure. As can be seen, the different parts of speech, represented by the numbers in the plot (1 is for articles, 2 for pronouns, the rest is unintelligible because of overlap), occupy frequency regions that seem to be of the right order of magnitude. But unlike in natural language, the different frequency bands do not line up. Also, the word classes form distinct groups, whereas in natural language, classes overlap (e.g. the most frequent N outranks the least frequent P by far). In sum, distinguishing between word classes does not suffice to explain Zipf’s law.

### Semantics

**Fig 3:**
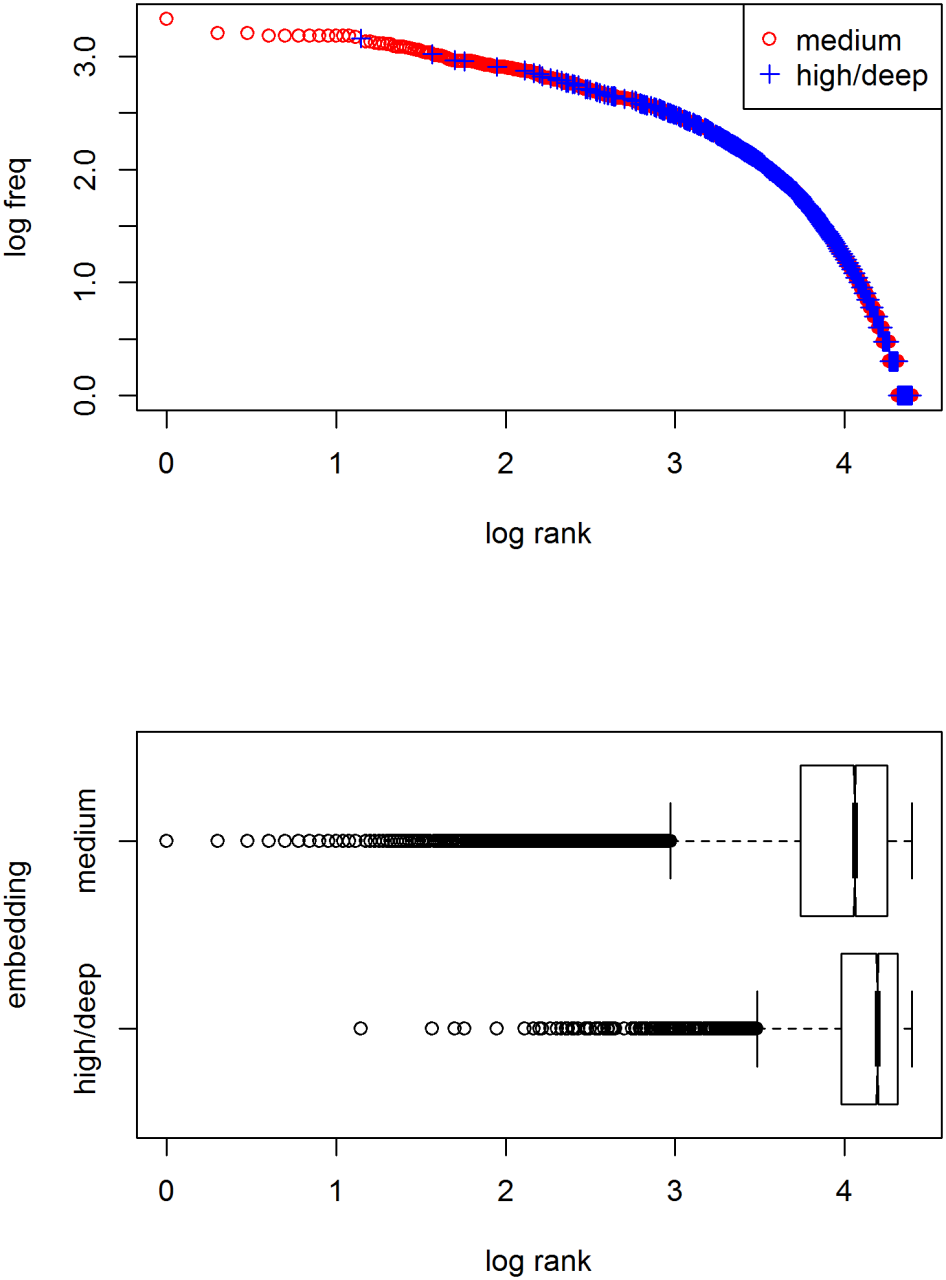
Frequency distributions of different specifity classes in the Brown corpus. Top panel: distribution over overall distribution of nouns. Degree of meaning specification is approximated by automatically determining the depth of embedding in the WordNet noun taxonomy. Words with lowest ranks are all moderately specified with an embedding of 3–9 (red circles). Bottom panel: boxplots of frequency ranks per specificity class.

As pointed out above, in order to become frequent, a word should be specific enough to single out its referent in context and general enough to be applied to different referents [11]. A simple way of approximating the degree of specification is by determining the depth of embedding of a word in a word taxonomy such as WordNet [12], assuming that a word inherits all of the specifications of its parent including those that set it apart from its sisters. (Note that this is only used as an initial proxy to show how meaning specificity matters; meaning will be operationalized differently in the remainder.) In WordNet, meanings are organized in synonym sets, groups of words with approximately the same meaning. Various lexical relations are determined between these sets. For our purposes, the most important relation is the *super–subordinate* or the *is-a* relation. For example, we find 17 subsequent superordinate sets for *submarine*, starting with *submersible, submersible warship*, and only two for *thing*, viz. *physical entity* ⊂ *entity*, the top node of the noun taxonomy. If we now look up the total frequency in the Brown corpus for all nouns in the two meaning sets, we find, not unexpectedly, that the latter is more frequent than the former (with 484 against 178 attestations, in which all 178 hits for submarine in fact were due to the synonym *sub*, which is homonymous and whose frequency is due to its other meaning *substitute*). (Note that this procedure does not distinguish within homonymic or polysemic sets, which is not a problem, as the simple word counts it tries to account for, such as the one in Fig 1, also ignore this.) We can check whether the intuition about the relation between meaning specificity/embedding depth and frequency of usage is right in general by doing the same for all nouns in WordNet. The top panel in Fig 3 shows the distribution of two different “specificity” classes over the overall frequency distribution of nouns in the Brown corpus, viz. nouns that have an embedding depth between 3 and 9 (*medium*; red circles), and nouns that are either on top or towards the lower ends of the taxonomy (*high/low*; blue pluses). Words that were not attested in the corpus were removed. As can be seen, the most frequently used concepts indeed are modestly specified with a depth of embedding of 3–9; that is, specific enough to be distinctive while general enough to be reusable. On the bottom panel, the distributions of the log rank per specificity class is shown. Words with modest specification have a lower rank (or higher frequency) on average and span the entire range; words with a high/low degree of specification have higher ranks only.

Instead of using embedding as an approximation, the degree of meaning specification of words can also be simulated, by generating an abstract lexicon in which words are specified for a number of meaning dimensions. The first dimension could be taken to represent a property that all concrete objects do and abstract objects do not have (i.e., it is activated in the vector representations of concrete objects only), the second dimension represents something animates objects do and inanimates do not have, etc. (cf. [13–16] for applications). Note that qualitatively, this is very different from the vector-semantics approach used in modern computational linguistics (e.g. [17, 18]), in which vectors represent behavior in texts rather than the underlying semantics that causes this behavior. Rather, the vectors used here should be understood as representations of activation in a neural-network model of the brain [19, 20].

Using this implementation, the usage of words is modeled by randomly generating contexts with a target object and a set of distractors that are fully specified for all meaning dimensions. Next, a word from the lexicon is selected that suffices to single out the target object. For example, we may have two words in our lexicon, the first of which, *a*, is specified for all three meaning dimensions, with values 0, 0, and 1 respectively, whereas the second, *b*, is specified for dimensions D1 and D3 only, with values 0 and 1 (cf. Table 2). If the target object is a 0 0 1, both words match in principle. In contexts with distractor objects that all happen to differ from the target on either D1 or D3 (the first four distractors in Table 2), both words *a* and *b* can be used; but whenever there is a distractor object that is similar to the target on both D1 and D3 (the fifth distractor), word *a* is necessary to uniquely refer to the target.

**Table 2:** Toy example of abstract lexicon and context. Words are specified for three dimensions or less, referential objects are always fully specified. To distinguish the target from the first four distractors, words *a* and *b* can both be used, in the presence of the fifth distractor, however, only *a* suffices.

word	D1	D2	D3
*a*	0	0	1
*b*	0	–	1
…			
target	0	0	1
distractors	1	0	1
	1	0	0
	1	1	1
	0	1	0
	*0*	*1*	*1*

**Fig 4:**
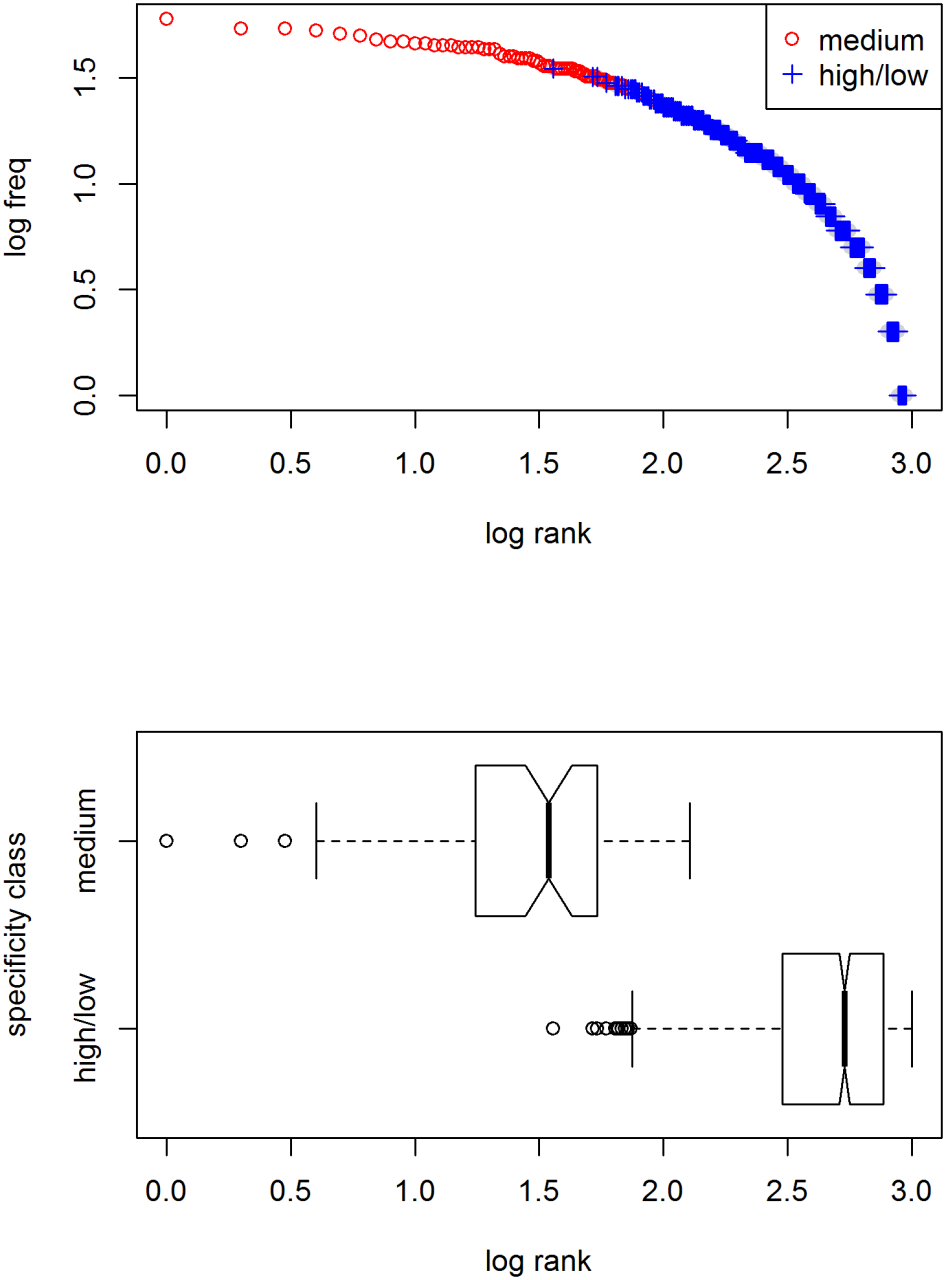
Frequency distribution of different specificity classes in a computer simulation. The lexicon consists of 1,000 words with ten optional meaning dimensions, from which words are selected for 10,000 contexts with randomly generated targets and 5 randomly generated distractors. Words with lowest ranks are all moderately specified (2–4 dimensions; red circles). Bottom panel: boxplots of frequency ranks per specificity class.

In a simulation whose results are shown in Fig 4, a lexicon of 1,000 words with ten meaning dimensions is used, from which words are selected for 10,000 contexts with randomly generated targets and 5 randomly generated distractors. As with the natural-language example in the previous figure, words of moderate specification are used most frequently.

**Fig 5:**
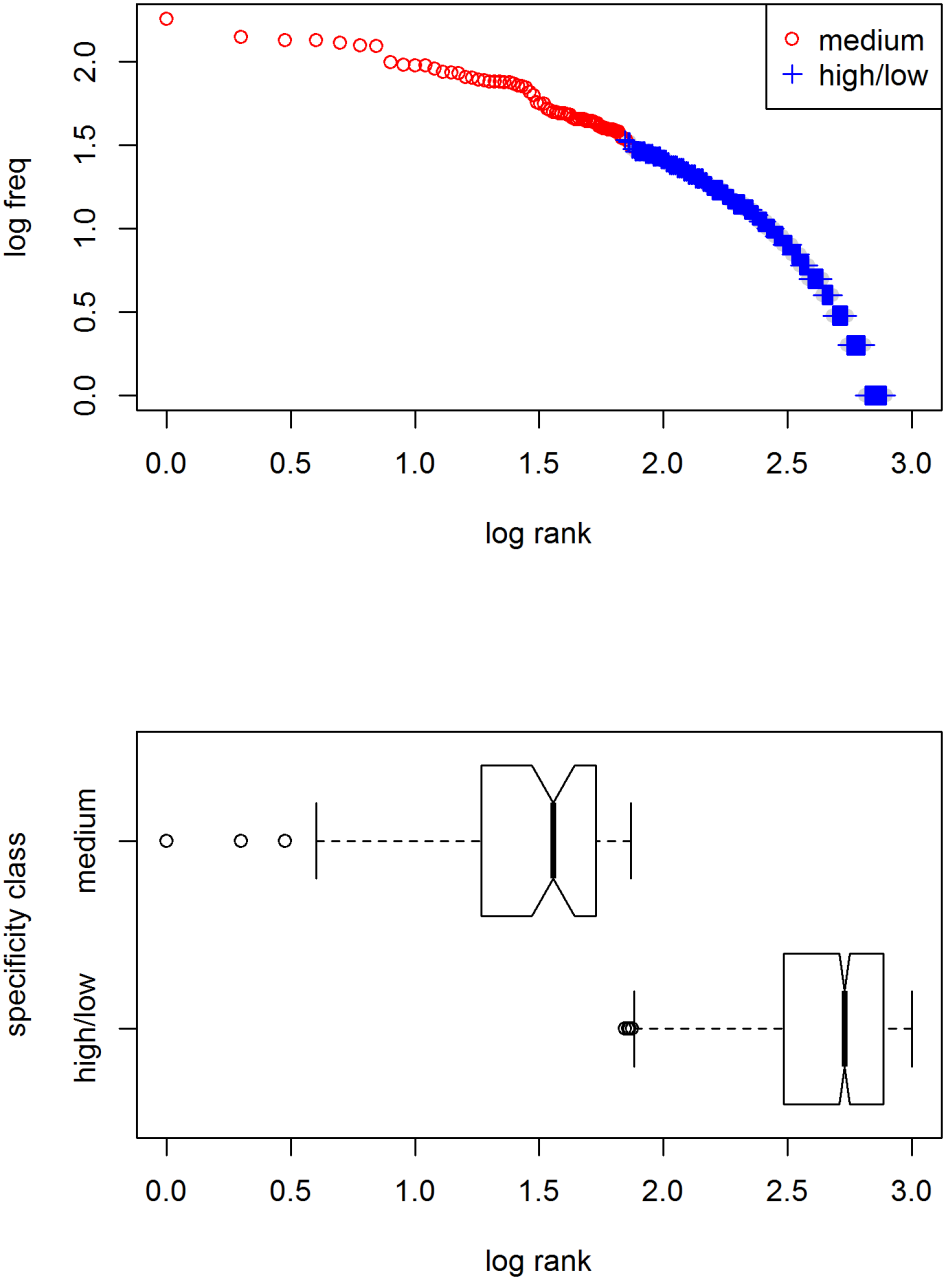
Distribution of probability of usage of different specificity classes in a computational model. The lexicon consists of 1,000 words with ten optional meaning dimensions. Probability of usage depends on degree of specification and number of distractors assumed (here 5). As in the previous figures, words with lowest ranks are all moderately specified (3–6 dimensions; red circles). Bottom panel: boxplots of frequency ranks per specificity class.

Given the match between the results from the combined WordNet/Brown study and the computer simulation, we can go one step further and develop a mathematical model of the dependence of usage frequency on degree of specification. Assuming binary meaning dimensions, the probability *p*_*a*_ that a word is applicable in principle is .5^*nDim*^, with *nDim* being the number of meaning dimensions for which that word is specified [21]. As this holds for both target and distractor objects alike, the probability that a word can actually be used in context is dependent on the number of distractor objects *n*: The probability *p*_*d*_ that there is no distractor object to which a word could apply is (1 − *p*_*a*_)^*n*^, hence the probability *p*_*u*_ that a word will be used is *p*_*a*_ * *p*_*d*_. We can now randomly generate words, assign them a degree of specification (without specifying the meaning dimensions), and calculate the expected usage frequency given a given number of distractor objects. The results are shown in Fig 5. The close similarity with the previous figure strongly suggest we have successfully modeled the interaction between meaning specification and usage frequency.

Importantly, as the results in Fig 3 already showed, semantics alone does not suffice to yield a Zipfian distribution: The frequency distribution within nouns is not the straight line through double-log space Zipf’s law prescribes.

## Combining syntax and semantics

**Fig 6:**
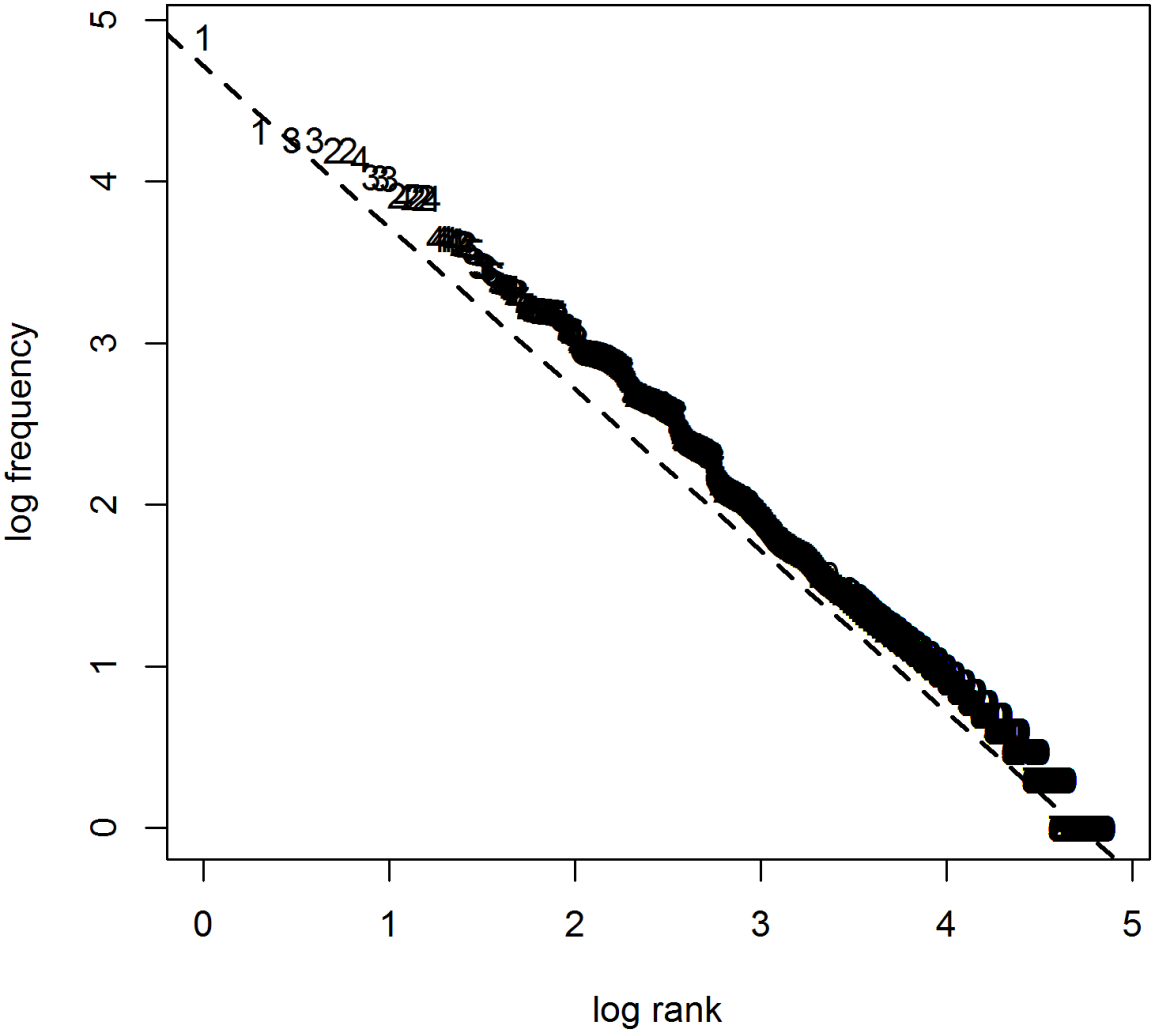
Generating Zipf’s law by combining syntax and semantics. 10 word classes of equal frequency are used with 5, 30, 50, 100, 500, 500, 1,000, 15,000, 25,000, and 100,000 members; items can be specified for maximally 30 meaning dimensions (mean 8.3, sd 2.0), and the number of distractors is 5.

In Fig 6, the results are shown when combining the two ingredients discussed above, using 10 word classes with 5, 30, 50, 100, 500, 500, 1,000, 15,000, 25,000, and 100,000 members, of equal frequency. The maximum number of dimensions an item is specified for is around 20 (given thirty optional meaning dimensions), and the number of distractors to calculate the usage probability is 5. As can be seen, the frequency development approximately follows Zipf’s law. Equally importantly, the frequency ranges of the different word classes overlap (although this is only visible for the lower ranks), just like in natural language.

**Fig 7:**
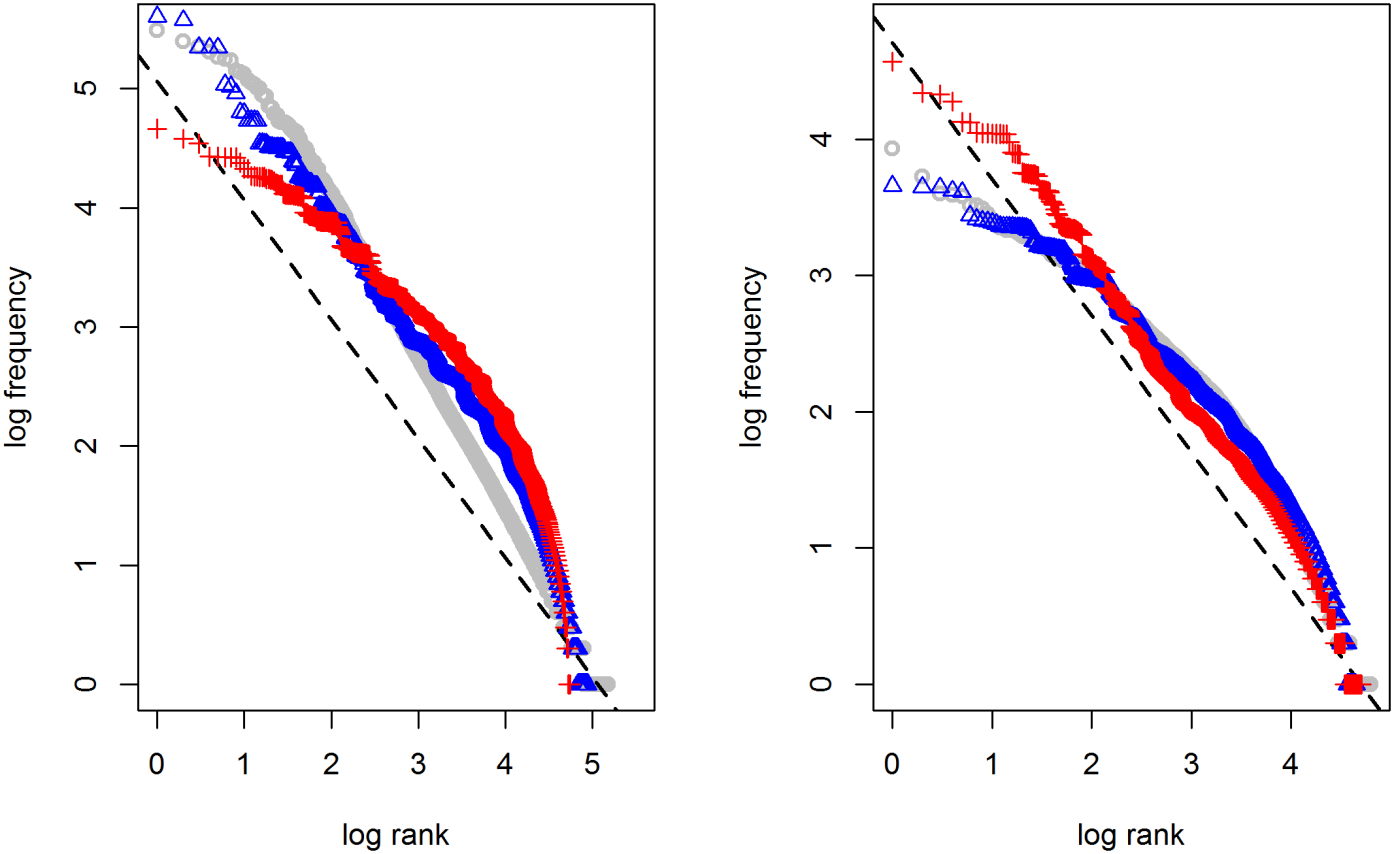
Frequency distribution in CGN (left) and Brown corpus (right). Blue triangles show the results of the model simulation using the corresponding parameters from Table 1; red plusses show the results when mixing the CGN and Brown parameters.

It has often been observed that natural language does not always behave neatly according to Zipf’s law (unlike Melville’s *Moby Dick* shown in Fig 1). Fig 7 illustrates that the frequency distributions in the CGN and Brown corpus, represented by the grey circles, deviate considerably from Zipf’s ideal. Interestingly, the model proposed here deviates from it in exactly the same way, but only if we use the corresponding class sizes and frequencies shown in Table 1 (blue triangles). If we mix the numbers, using the CGN class parameters to simulate a corpus of the Brown size and the other way around, there is no match (red pluses).

## Discussion

In this paper I have shown how a Zipfian distribution can be explained by the interaction of syntax and semantics, thus providing a linguistically informed explanation of Zipf’s law. Words are from different parts-of-speech classes, which differ in size by orders or magnitude. Within classes, words differ in meaning by being differentially specified for a number of meaning dimensions. If a word is specified for a few dimensions only, it becomes ambiguous; if it is overly specific, it will hardly ever be applicable. It was shown that neither of these ingredients suffices to produce Zipf’s law, but together they can.

Where the results differ from the Zipfian ideal, they do so in the way natural language does. Thus, the model does not “overfit” Zipf’s law but really seems to capture the underlying language mechanisms that drive it. This is all the more important as there are many ways of deriving a Zipfian distribution [22], whereas the real interest is of course in the natural-language phenomenon.

According to Piantadosi, a “[t]rue psychological account” of Zipf’s law should be based on independently testable phenomena and mechanisms that fit with known psychological processes of word production and language use [7]. One should thus not only derive the law but motivate the underlying assumptions. By having provided independent evidence for the ingredients (the most frequently used words were shown to be moderately specified and POS differences were established for three different languages), the ecological validity of the proposed mechanisms should be clear. It is equally important to show that this does not hold for some of the competing proposals. In S2 Text, a number of models are discussed that more or less span the range from explaining Zipf’s law as a statistical quirk [23–25] to understanding it as the inherent result of a communication system [6, 21, 26–29]. For a more elaborate review, I refer the interested reader to the review by Piantadosi, who (indeed) concludes that many proposals focus on the simple law rather than the less simple data and do not account for psychological processes of language production [7]. I hope to have shown that neither of these concern applies to my proposal.

Two puzzles still remain to be explained: Why a slope of –1 in double log space and what about Zipf’s law in other, non-linguistic domains, where it is often reported too? Starting with the latter, such distributions in fact seem to “suffer from a lack of sufficient statistics in the region corresponding to the high values of the rank variable”, according to Montemurro [3]. He claims that Zipf’s law only applies to the first subset of the measurements; for the remainder of the data, a new power regime holds (the parameters of which depend on corpus size). Also Newman observes that “[f]ew real-world distributions follow [the same] power law over their entire range, and in particular not for smaller values of the variable being measured” [22]. Crucially, this means that Zipf’s law need not be a “universal law for complex systems” [30] but that a language-specific explanation may be appropriate indeed. As for the first question, the distinctive slope can be explained as an accidental result of the relative sizes of word classes: It follows logically from open classes being magnitudes of orders larger in size than closed classes (cf. S1 Text for formal proof).

## Conclusion

Zipf’s law can be explained through the interaction between syntax and semantics. By using these ingredients, the ecological validity of the model is warranted. Moreover, the model predictions deviate from the Zipfian ideal in exactly the same way as natural language does.

## Supporting information

S1 File Scripts. (R)

S2 File Data. (R)

S3 File Alternative models. (R)

S1 Text Derivation of the slope. (PDF)

S2 Text Alternative models. (PDF)

S1 Fig Simulation of Miller’s proposal. (PDF)

S2 Fig Mandelbrot’s law. (PDF)

S3 Fig Results for replication of Jäger and van Rooij. (PDF)

S4 Fig Results for replication of Manin. (PDF)

## References

[1] Zipf GK. Human behavior and the principle of least effort An introduction to human ecology. New York and London: Hafner publishing company; 1949.

[2] Mitzenmacher M. A brief history of generative models for power law and lognormal distributions. Internet mathematics. 2004;1(2):226–251. 10.1080/15427951.2004.10129088

[3] Montemurro MA. Beyond the Zipf-Mandelbrot law in quantitative linguistics. Physica A. 2001;300:567–578. 10.1016/S0378-4371(01)00355-7

[4] Pustet R. Zipf and his heirs. Language Sciences. 2004;26:1–25. 10.1016/S0388-0001(03)00018-4

[5] Kello CT, Brown GDA, Ferrer-i-Cancho R, Golden JG, Linkenkaer-Hansen K, Rhodes T, et al Scaling laws in cognitive sciences. Trends in Cognitive Sciences. 2010;14(5):223–232. 10.1016/j.tics.2010.02.005 20363176

[6] Manin DY. Zipf’s Law and avoidance of excessive synonmy. Cognitive Science. 2008;32:1075–1098. 10.1080/03640210802020003 21585444

[7] Piantadosi ST. Zipf’s word frequency law in natural language: A critical review and future directions. Psychonomic Bulletin & Review. 2014;21:1112–1130. 10.3758/s13423-014-0585-6 24664880 PMC4176592

[8] Oostdijk N. The Spoken Dutch Corpus. Overview and First Evaluation. In: Proceedings of the 2nd International Conference on Language Resources & Evaluation; 2000.

[9] Francis WN, Kučera H. A Standard Corpus of Present-Day Edited American English, for use with Digital Computers (Brown). Providence, Rhode Island: Brown University; 1964, 1971, 1979.

[10] Oravecz C, Váradi T, Sass B. The Hungarian Gigaword Corpus. In: Proceedings of LREC 2014; 2014. p. 1719–1723.

[11] Rosch E. Principles of categorization In: Rosch E, Lloyd BB, editors. Cognition and categorization. Hillsdale, New Jersey: Lawrence Erlbaum; 1978 p. 27–48.

[12] Princeton University. WordNet; 2010. Available from: http://wordnet.princeton.edu.

[13] Lestrade, S. Simulating the development of bound person marking. In: Baayen, H, Jäger, G, Köllner, M, Wahle, J, Baayen-Oudshoorn T, editors. Proceedings of the 6th Conference on Quantitative Investigations in Theoretical Linguistics. Tuebingen: University of Tuebingen; 2015.

[14] Lestrade S. The Emergence Of Argument Marking. In: Roberts SG, Cuskley C, McCrohon L, Barceló-Coblijn L, Fehér O, Verhoef T, editors. The Evolution of Language: Proceedings of the 11th International Conference (EVOLANG11); 2016. Available from: http://evolang.org/neworleans/papers/36.html.

[15] Lestrade S. A case of cultural evolution: The emergence of morphological case. Linguistics in the Netherlands. 2015;32:105–115. 10.1075/avt.32.08les

[16] Lestrade S. The emergence of differential case marking In: Seržant IA, Witzlack-Makarevich A, Mann K, editors. The Diachronic Typology of Differential Argument Marking. Language Science Press; (to appear).

[17] Deerwester S, Dumais ST, Furnas GW, Landauer TK, Harshman R. Indexing by latent semantic analysis. Journal of the American Society for Information Science. 1990;41(6):391–407. 10.1002/(SICI)1097-4571(199009)41:6<391::AID-ASI1>3.0.CO;2-9

[18] Mikolov T, Chen K, Corrado G, Dean J. Efficient Estimation of Word Representations in Vector Space; 2013. ArXiv:1301.3781 [cs.CL].

[19] Rumelhart DE, McClelland JL, the PDP Research Group. Parallel distributed processing: Explorations in the microstructure of cognition. Cambridge, Mass: MIT Press; 1986.

[20] Paul Smolensky GL. The harmonic mind: From neural computation to optimality-theoretic grammar. Cambridge, Mass: MIT Press; 2006.

[21] Guiraud P. The semic matrices of meaning. Social science information. 1968;7(2):131–139. 10.1177/053901846800700206

[22] Newman MEJ. Power laws, Pareto distributions and Zipf’s law. Contemporary physics. 2005;46(5):323–351. Available from: arxiv.org/pdf/cond-mat/0412004. 10.1080/00107510500052444

[23] Miller GA. Some effects of intermittent silence. The American Journal of Psychology. 1957;70:311–314. 10.2307/1419346 13424784

[24] Howes D. Zipf’s Law and Miller’s Random-Monkey Model. The American Journal of Psychology. 1968;81(2):269–272. 10.2307/1421275

[25] Conrad B, Mitzenmacher M. Power Laws for Monkeys Typing Randomly: The Case of Unequal Probabilities. IEEE Transactions on information theory. 2004;50(7):1403–1414. 10.1109/TIT.2004.830752

[26] Mandelbrot B. An informational theory of the statistical structure of languages. In: Jackson W, editor. Communication Theory. Betterworth; 1953 p. 486–500.

[27] Ferrer i Cancho R, Solé RV. Least effort and the origins of scaling in human language. PNAS. 2003;100(3):788–791. 10.1073/pnas.0335980100 12540826 PMC298679

[28] Tullo C, Hurford JR. Modelling Zipfian Distributions in Language; 2003. Paper presented at the Language Evolution and Computation Workshop/Course at the 15th European Summer School on Logic Language and Information, Vienna.

[29] Jäger G, van Rooij R. Language structure: Psychological and social constraints. Synthese. 2007;159(1):99–130. 10.1007/s11229-006-9073-5

[30] Silagadze ZK. Citations and the Zipf-Mandelbrot Law. Complex Systems. 1997;11:487–499.

[31] R Core Team. R: A Language and Environment for Statistical Computing. Vienna, Austria; 2012 Available from: http://www.R-project.org/.

