# Supplementary material for: Unzipping Zipf’s law
Source: PLoS One. 2017 Aug 9;12(8):e0181987. doi: 10.1371/journal.pone.0181987 (PMC5549924; doi:10.1371/journal.pone.0181987)
Supplement: S1 Text — (PDF) [file pone.0181987.s004.pdf]

Derivation of the slope

The explanation for the slope puzzle is largely in the relative sizes of classes. The average frequency of the smallest closed class $mean(freq_C)$ can be calculated by dividing the total class frequency $total_C$ by the class size $size_C$ (as for any other class). If there is to be a straight line with slope –1 through double-log space,

(1) a. $log(freq(rank_i)) = log(max(freq)) - log(rank_i)$,
b. $log(max(freq)) = log(max(rank))$, and therefore
c. $log(freq(rank_i)) = log(max(rank)) - log(rank_i)$.

For class C, the smallest closed class including the most frequently used item, it should also hold that

(2) $log(mean(freq_C)) = log(max(rank)) - .5 * log(size_C)$.

That is, given a slope of –1 (in an equidistribution), the mean of a class should correspond to its median value.

Given our initial observation about $mean(freq_C)$, we have

(3) $log(total_C/size_C) = log(max(rank)) - .5 * log(size_C)$,

which can be rewritten as

(4) a. $log(total_C) - log(size_C) = log(max(rank)) - .5 * log(size_C)$
b. $.5 * log(size_C) = log(total_C) - log(max(rank))$.

$Total_C$ is proportional to the corpus size and follows from the grammar:

(5) a. $total_C = proportion_C * corpusSize$
b. $log(total_C) = log(corpusSize) + log(proportion_C)$.

Combining (4-b) and (5-b), we get

(6) $.5 * log(size_C) = log(corpusSize) + log(proportion_C) - log(max(rank))$,

or equivalently

(7) $.5 * log(size_C) - log(proportion_C) = log(corpusSize) - log(max(rank))$.

Undoing the logarithms, this means that

(8) $\frac{\sqrt{size_C}}{proportion_C} = \frac{corpusSize}{max(rank)}$

Since S is a closed class, $size_C$ does not increase with corpus size (once all members are attested, that is, which should hold for relatively small corpora already). Also $proportion_C$ is a given, as it follows from the grammar. For example, if articles are obligatory, you have to use an article for each noun. Hence the left hand side of the equation can be considered a constant. Since the maximum rank, i.e. the number of types, can only increase through the open classes, the combined open class sizes $size_O$ should grow proportionally with corpus size. Finally, if we consider the fact that $max(rank)$ is the sum of $size_C$ and $size_O$, we get:

(9) a. $\frac{\sqrt{size_C}}{proportion_C} = \frac{corpusSize}{size_O + size_C}$
b. $size_O = \frac{corpusSize}{\sqrt{size_C}/proportion_C} - size_C$

For Melville's *Moby Dick*, which was shown in the main text to come close to the Zipfian ideal, (9-b) predicts an open class size of approximately 11,269 types (sum

proportion of three articles is .09 and the total length is 216,926 words):

(10) $$size_O = \frac{216{,}926}{\sqrt{3}/.09} - 3 = 11,269$$

This estimation is in the right ballpark given the attested number of types of 17,507. More generally, assuming a class size of four and class frequency of .1 for class C as a rule of thumb, (9) predicts that the joint size of the open classes should be the corpus size divided by 20, which seems reasonable.

In sum, for a slope of −1 in double-log space and Zipf's law to be applicable, the combined size of the open classes should exceed that of the closed classes by several orders of magnitude.
