# Supplementary material for: Unzipping Zipf’s law
Source: PLoS One. 2017 Aug 9;12(8):e0181987. doi: 10.1371/journal.pone.0181987 (PMC5549924; doi:10.1371/journal.pone.0181987)
Supplement: S2 Text — (PDF) [file pone.0181987.s005.pdf]

Alternative models

As mentioned in the introduction of this paper, there is a wide variety of proposals for explaining Zipf's law. In this section, a number of models are discussed that more or less span the range from explaining Zipf's law as a statistical quirk [1–3] to understanding it as the inherent result of a communication system [4–9].

Miller argues that a Zipfian distribution can emerge from (monkeys) randomly typing on a keyboard [1]. This would yield a corpus of words with various length, in which increasingly long words are increasingly less frequent, as accidentally hitting the space bar must be avoided longer. The results of a random typing experiment are shown on the A panel of S1 Figure. The dashed diagonal shows the corresponding perfect Zipfian distribution, with a slope of -1 and going through the mean rank of the elements sharing the lowest frequency. As can be seen, the simple version of Miller's proposal cannot account for the frequency development of most frequent words, or, more generally, for frequency differences within length classes. As shown on panel B, the results improve significantly if character probability is taken into account (character probabilities are estimated on the basis of *Moby Dick*).

Miller's proposal is sometimes considered a valid argument for a shallow linguistic basis of Zipf's law [2, 3]. Now we could use Occam's razor and prefer random typing as the simplest account possible over more intricate procedures, but note that the predicted abstract frequencies clearly fall short of the Zipfian ideal in Panel B. Moreover, actual word rank or frequency cannot be predicted on the basis of the probability that follows from their constituting characters: In Panel C, the correlation is shown between the actual frequency rank of the words in *Moby Dick* and the one predicted on the basis of their Miller probabilities (e.g., the 281st most frequent word *moby* has a Miller probability of $p(m) * p(o) * p(b) * p(y) = .020 * .059 * .014 * .014 = 2.3 * 10^{-7}$ on the basis of which it should have rank 2391). And even if these results came close, which they clearly don't, we should be concerned about the ecological validity of the mechanisms involved. As Newman notes, "there are many different mechanisms for producing power laws and [...] different ones are applicable to different cases" [10]. That is, if we want to explain word frequencies, we cannot ignore syntax and semantics.

Differently from Miller, Mandelbrot prefers a linguistic foundation, complaining that Zipf's work "was not based in the least on any serious linguistic theory, nor on communication theory" [4]. Using the linguistic ideas of de Saussure, he considers language as "a random sequence of concrete entities". Mandelbrot argues that a Zipf curve emerges if a balance is found between informativity (in terms of Shannon's *entropy*, the inverse log of frequency) and production costs (as measured in word length). The frequency p_n of the ith entity is said to be better described by the formula $p_i = P(i + m)^{-B}$, in which P, m, and B are positive constants. The additional parameters m and B improve the description for the lower and higher ranks, respectively. Conceptually, m should represent the richness of the coding system (e.g., the number of letters in the alphabet; the smaller m, the more symbols available), B depends (non-linearly) on the average informativity of a word. Technically, B determines the slope of the development in double-log space, m the degree to which it is bent. In many natural-language texts the frequency development of the lower ranks indeed deviates somewhat from Zipf's ideal and can be captured better by Mandelbrot's law, as illustrated in S2 Figure, in which panel A shows Herman Melville's *Moby Dick*, panel B Jane Austen's *Sense and Sensibility*, panel C Mark Twain's *Adventures of Huckleberry Finn*, and panel D, finally, James Joyce' *Ulysses*.

According to Mandelbrot, Zipf's law is the "particular case" in which $B = 1$ and $m = 0$. The Mandelbrot parameters for the texts in S2 Figure are given in Table 1, in which B is calculated by the least-squares method and m is determined heuristically. Note that it is unclear why and how these parameters should differ as they do between

these texts. Another objection that can be made against Mandelbrot's proposal is that people don't just use words to be informative, but rather to refer to things they want to talk about ([6,11]; note that the same objection could be made against Miller previously, *mutatis mutandis*). Thirdly, nothing in Mandelbrot's proposal explains *why* the top most frequently used words consists of exactly these words, nor why they should differ in order between authors.

panel	text	length	P	B	m	top 5
A	*MD*	$10^{4.16}$	$10^{4.79}$	1.15	5	*the, of, and, a, to*
B	*SaS*	$10^{3.75}$	$10^{5.05}$	1.34	11	*to, the, of, and, her*
C	*HF*	$10^{3.73}$	$10^{4.91}$	1.32	8	*and, the, I, a, to*
D	*U*	$10^{4.34}$	$10^{4.45}$	1.03	1	*the, of, and, a, to*

Table 1. Mandelbrot parameters and top 5 for texts in S2 Figure. MD: *Moby Dick*, SaS: *Sense and Sensibility*, HF: *The adventures of Huckleberry Finn*, U: *Ulysses.* B is calculated by the least-squares method and m is determined heuristically.

A third, more general type of explanations for Zipfian distributions makes use of *preferential-attachment* ([12]; cf. [13] for a discussion of more recent work along these lines). In general, the idea is that what is frequent becomes more frequent, as the selection of entities is partly dependent on their selection history. In language, some words indeed are more frequent in some contexts than in other contexts for reasons of topic coherence. Jäger and van Rooij model this intuition by sampling a sequence of 100 words from a lexicon of 100,000 words ([9]; cf. [8] for a similar attempt). Next, with a probability of 1/8, a word is sampled from the lexicon and added to the sequence (in which case a new topic is started); alternatively, with a probability of 7/8, a word is drawn from the last 100 words in the sequence (i.e., the topic is continued). After repeating this procedure 100,000 times, a frequency distribution emerges that can be described by Mandelbrot's formula, using $P = 10^{4.6}, B = 1.1, m = 100$ (cf. S3 Figure). Jäger and van Rooij conclude on the basis of their results that Zipf's law is an invariant of language usage, and does not say anything about its structure [9]. This seems much too bold a claim, however. First, the most frequent items in their model fall short. Whereas in natural language, the logged frequency of the most frequent item roughly corresponds to the logged rank of the least frequent one, the most frequent item in their model reaches less than three-fourths of this (because of which their m parameter is of a different order of magnitude than for the natural-language texts above). Again, Zipf's law is a special type of power law, viz. one in which the exponent is –1 because of which the slope of the development in double log-space is 45°; it is relatively easily to come up with a power law explanation, but getting the numbers right turns out to be quite hard. Secondly, if we take a closer look "inside" their distribution, we find that the words that are most frequent in the first part of the sequence and those that are most frequent in the last part form two completely disjoint sets. Whereas this could be expected of the usage of specific content words indeed, as topics change over time, this is of course not what happens in natural language in general: There, it is the same set of words that is most frequent throughout the entire discourse (cf. e.g. the top 5 in Table 1).

Following a fourth type of reasoning, Guiraud argues that Zipf's law must be determined by meaning (independently from context; [5]). He proposes semantics is a system of discrete "semes", organized in binary pairs such as animate-inanimate, actor-process. Words are combinations of semes, and the number of semes that are combined determines the frequency of a word: the more semes, the less frequent (for if semes have probability q, the probability of using a word combining s semes is q^s; cf. Table 2). There are a number of problems with the proposal of Guiraud. First, if this were all to it, *vertebrate* should be much more frequent than *dog*, as being a hypernym of the latter, it combines less semes by definition. This prediction does not seem to be

right (and at least in terms of internet query results, this is clearly not the case indeed.) Instead, there seems to be a preferred level of granularity, which is mostly independent of context ([14]). That is, the competition is not just between "overlapping" words of different specificity levels, but mostly between mutually exclusive words (*cat* vs *dog*). Second, as noted by [6], if semes were equiprobable, they should be orthogonal, but some of the examples suggest an implicative relation (e.g., being masculine/feminine strongly suggests being animate, or generally is irrelevant for inanimates, semantically at least) whereas others clearly exclude each other (e.g. processes cannot be animate). Thirdly, it can be expected that the probability of semes differs, some dimensions (and some values on some dimensions) being intrinsically more salient than others.

Table 2. First entries of a Guiraud lexicon.

ID	prob	V1	V2	V3	V4	V5	V6	V7
1	.02	0	1	1	0	0		1
2	.12		0			1	0	
3	.02	1	0	0		1	0	1
4	.12	0	1					0
5	.06	1		0		0	0	
6	.02		0	0	0	0	1	1

The probability for a word is calculated using a q of 5.

Varying on Guiraud's idea, Manin proposes that the world is conceptualized in layers of increasing specificity [6]. Semes in this proposal, although not explicitly named as such, are not independent, but bisect semantic subspaces into ever increasing detail. The most general word covers everything, the next two words cover the two halves, the next four words each take care of a quarter of semantic space, etc. This *Zipfian covering* can be shown to develop automatically from words increasing in meaning scope, until and unless colliding with words with a similar scope (i.e., synonymy is avoided; [6]). If one now assumes that the frequency of a word is proportional to its meaning extent, a Zipfian distribution is said to follow automatically. In fact, this is not quite true. As can be seen in S4 Figure, the resulting frequency distribution is bent the wrong way and decreases much too slowly. (In the original figure, the two axes are not equivalent because of which the fit seems better; see [6], p. 1084.) Also, although it may solve Guiraud's orthogonality problem, it still suffers from the others: There is an overall preferred level of granularity and some meanings may inherently be more frequent than others of the same specificity.

Finally, Ferrer i Cancho and Solé show how Zipf's original proposal in terms of the principle of least effort can be made explicit (and proven formally) using referential homonymy [7]. Glossing over the mathematical details, if the object space to refer to becomes too large, it is best to introduce some homonymy in the system as the lexicon would otherwise explode. The first problem with this account is conceptual, as I'm not sure whether the hearer would suffer from a large lexicon and whether the speaker would always want maximal explicitness. The size and organization of the lexicon could also, and better, it seems, be accounted for using learnability constraints (such as Kirby's *learning bottleneck*; [15]), which hold for speaker and hearer alike. More problematic, however, is the assumption that all words are said to be referential, whereas, in fact, the most frequent words are not, as we have seen already in Table 1. (Cf. [16] for more elaborate discussion.)

References

1. Miller GA. Some effects of intermittent silence. The American Journal of Psychology. 1957;70:311–314.
2. Howes D. Zipf's Law and Miller's Random-Monkey Model. The American Journal of Psychology. 1968;81(2):269–272.
3. Conrad B, Mitzenmacher M. Power Laws for Monkeys Typing Randomly: The Case of Unequal Probabilities. IEEE Transactions on information theory. 2004;50(7):1403–1414.
4. Mandelbrot B. An informational theory of the statistical structure of languages. In: Jackson W, editor. Communication Theory. Betterworth; 1953. p. 486–500.
5. Guiraud P. The semic matrices of meaning. Social science information. 1968;7(2):131–139.
6. Manin DY. Zipf's Law and avoidance of excessive synonmy. Cognitive Science. 2008;32:1075–1098.
7. Ferrer i Cancho R, Solé RV. Least effort and the origins of scaling in human language. PNAS. 2003;100(3):788–791.
8. Tullo C, Hurford JR. Modelling Zipfian Distributions in Language; 2003. Paper presented at the Language Evolution and Computation Workshop/Course at the 15th European Summer School on Logic Language and Information, Vienna.
9. Jäger G, van Rooij R. Language structure: Psychological and social constraints. Synthese. 2007;159(1):99–130.
10. Newman MEJ. Power laws, Pareto distributions and Zipf's law. Contemporary physics. 2005;46(5):323–351. Available from: `arxiv.org/pdf/cond-mat/0412004`.
11. Ross ASC. Discussion of "An informational theory of the statistical structure of languages". In: Jackson W, editor. Communication Theory. Betterworth; 1953. p. 500–501.
12. Simon HA. On a class of skew distribution functions. Biometrika. 1955;42(3-4):425–440.
13. Mitzenmacher M. A brief history of generative models for power law and lognormal distributions. Internet mathematics. 2004;1(2):226–251.
14. Rosch E. Principles of categorization. In: Rosch E, Lloyd BB, editors. Cognition and categorization. Hillsdale, New Jersey: Lawrence Erlbaum; 1978. p. 27–48.
15. Kirby S. Learning, bottlenecks and the evolution of recursive syntax. In: Briscoe T, editor. Linguistic Evolution Through Language Acquisition. Cambridge: Cambridge University Press; 2002. p. 173–204.
16. Piantadosi ST. Zipf's word frequency law in natural language: A critical review and future directions. Psychonomic Bulletin & Review. 2014;21:1112–1130.
