# Supplementary material for: Unzipping Zipf’s law
Source: PLoS One. 2017 Aug 9;12(8):e0181987. doi: 10.1371/journal.pone.0181987 (PMC5549924; doi:10.1371/journal.pone.0181987)
Supplement: S1 Fig — (PDF) [file pone.0181987.s006.pdf]

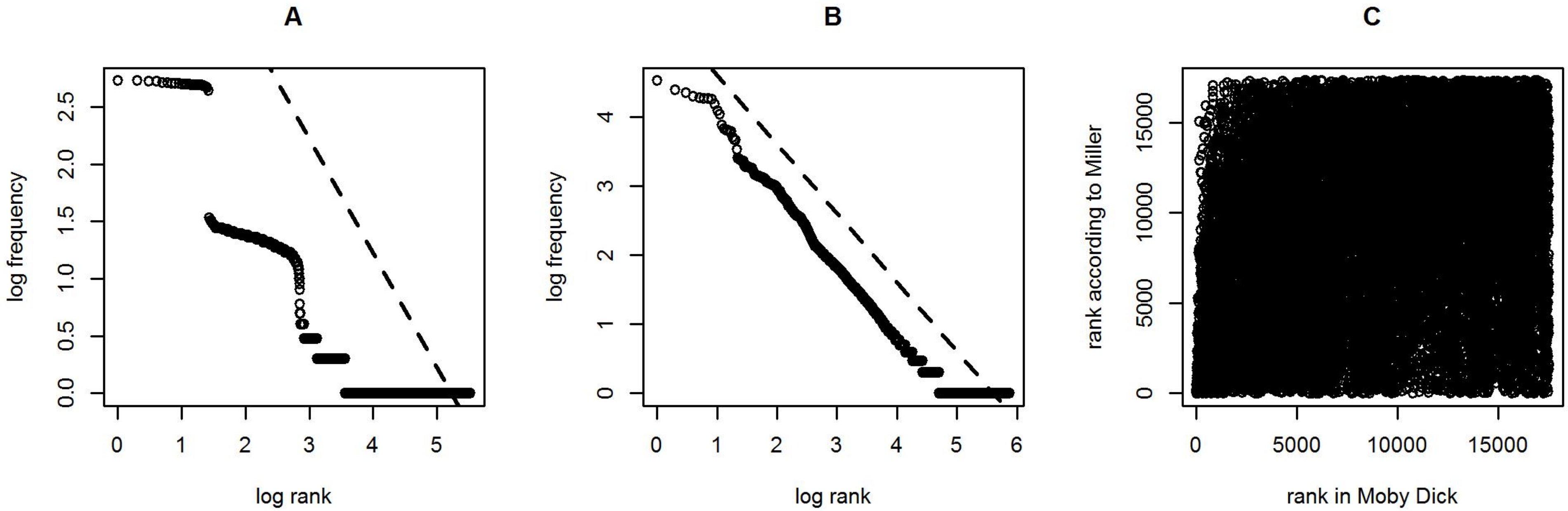

Simulation of Miller's proposal. A: predicted frequency by rank in double-log space with uniform character probability. B: using differential character probability (as determined by *Moby Dick*). Dashed lines show ideal Zipfian distribution. C: rank of words in *Moby Dick* against their rank as predicted by Miller.
