# Supplementary material for: Unzipping Zipf’s law
Source: PLoS One. 2017 Aug 9;12(8):e0181987. doi: 10.1371/journal.pone.0181987 (PMC5549924; doi:10.1371/journal.pone.0181987)
Supplement: S2 Fig — (PDF) [file pone.0181987.s007.pdf]

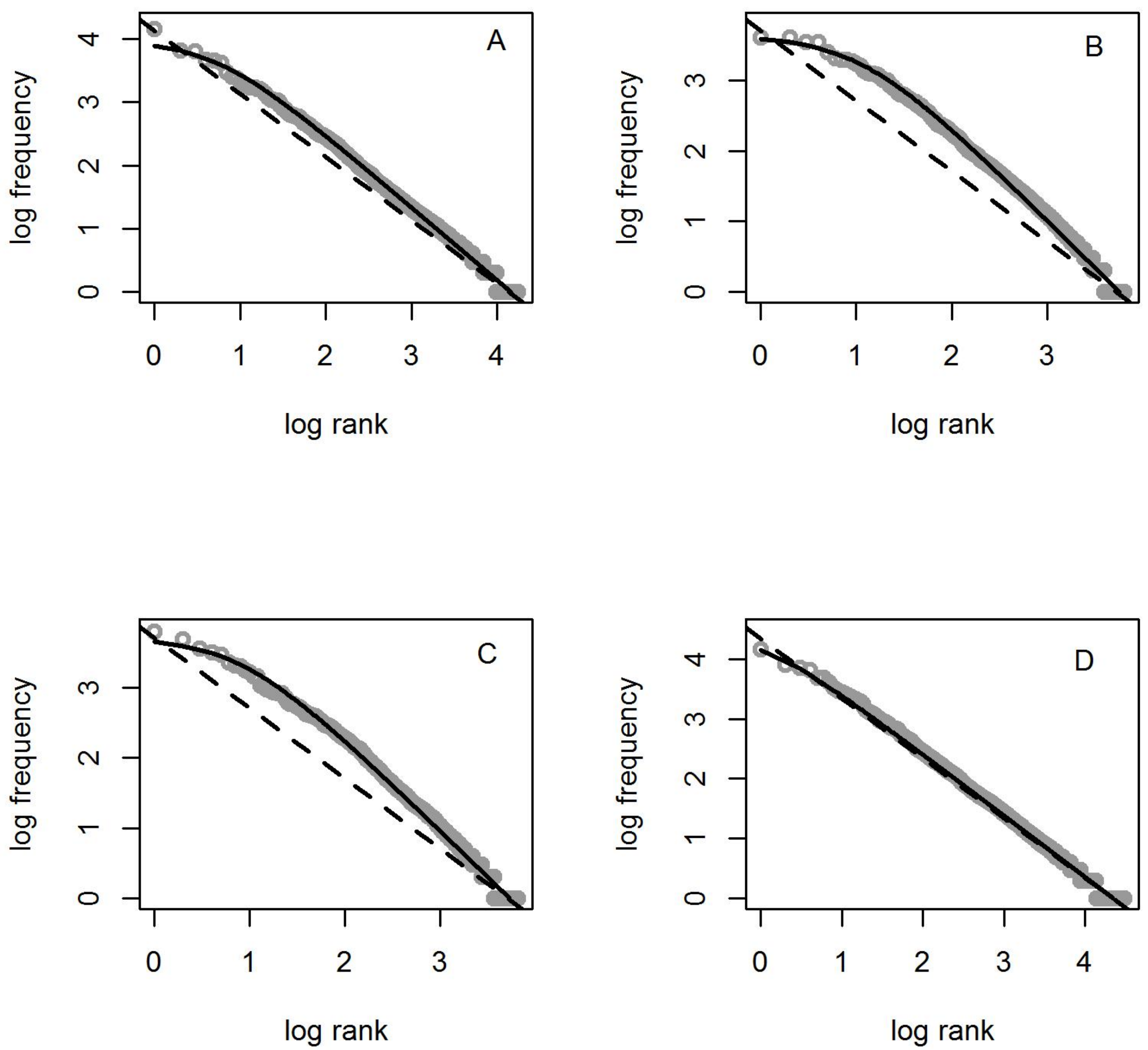

Mandelbrot's law. Model parameters are given in Table 1 of S2 Text. A: Herman Melville's *Moby Dick*. B: Jane Austen's *Sense and Sensibility*. C: Mark Twain's *Adventures of Huckleberry Finn*. D: James Joyce' *Ulysses*.
