# Supplementary material for: Unzipping Zipf’s law
Source: PLoS One. 2017 Aug 9;12(8):e0181987. doi: 10.1371/journal.pone.0181987 (PMC5549924; doi:10.1371/journal.pone.0181987)
Supplement: S3 Fig — (PDF) [file pone.0181987.s008.pdf]

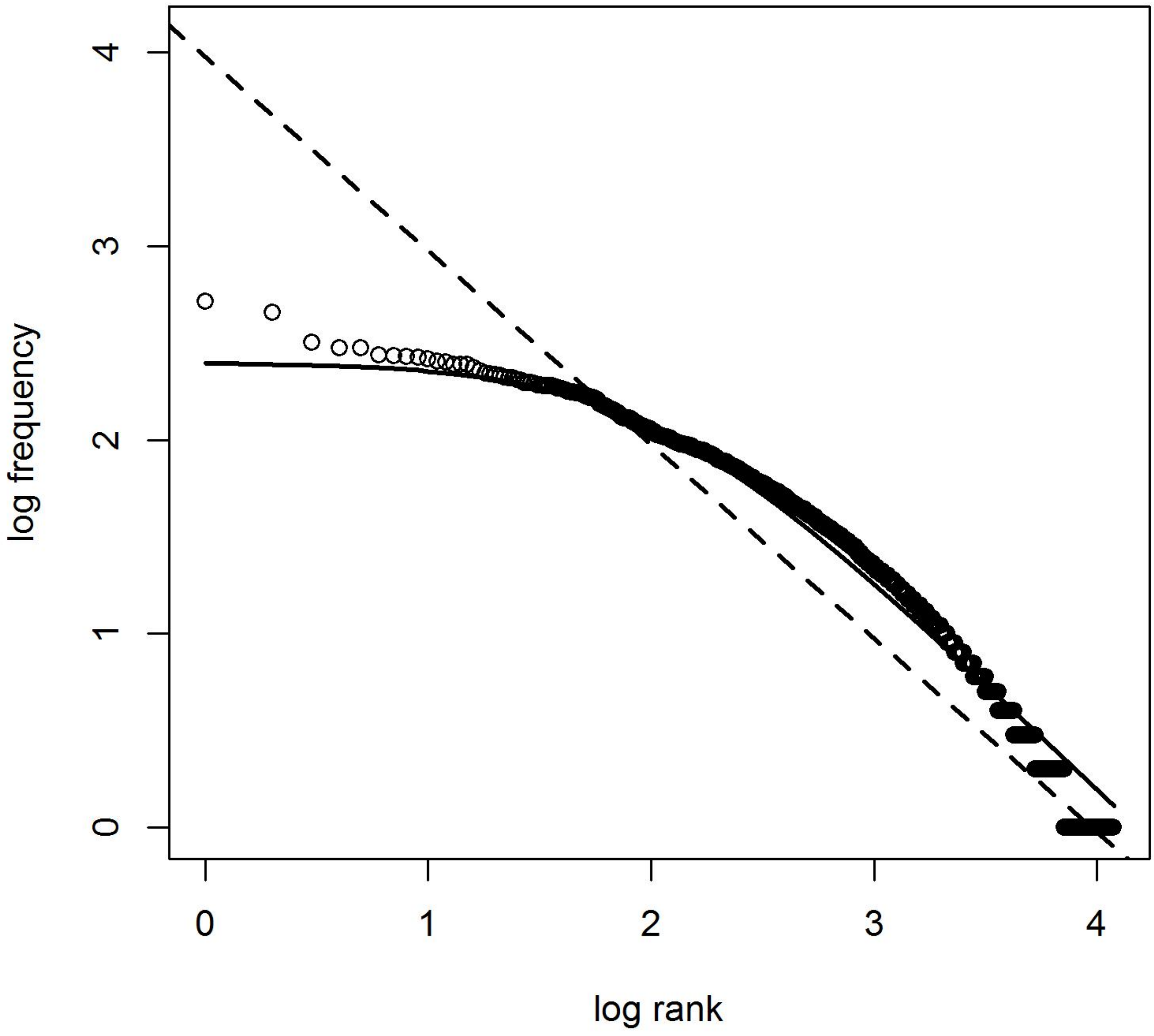

Results for replication of Jäger and van Rooij. Dashed lines show ideal Zipfian distribution, solid line Mandelbrot's law using originally suggested parameters: P = 104.6, B = 1.1, m = 100, and n = 12,000.
