# Supplementary figures and images for: Unzipping Zipf’s law
Source: PLoS One. 2017 Aug 9;12(8):e0181987. doi: 10.1371/journal.pone.0181987 (PMC5549924; doi:10.1371/journal.pone.0181987)

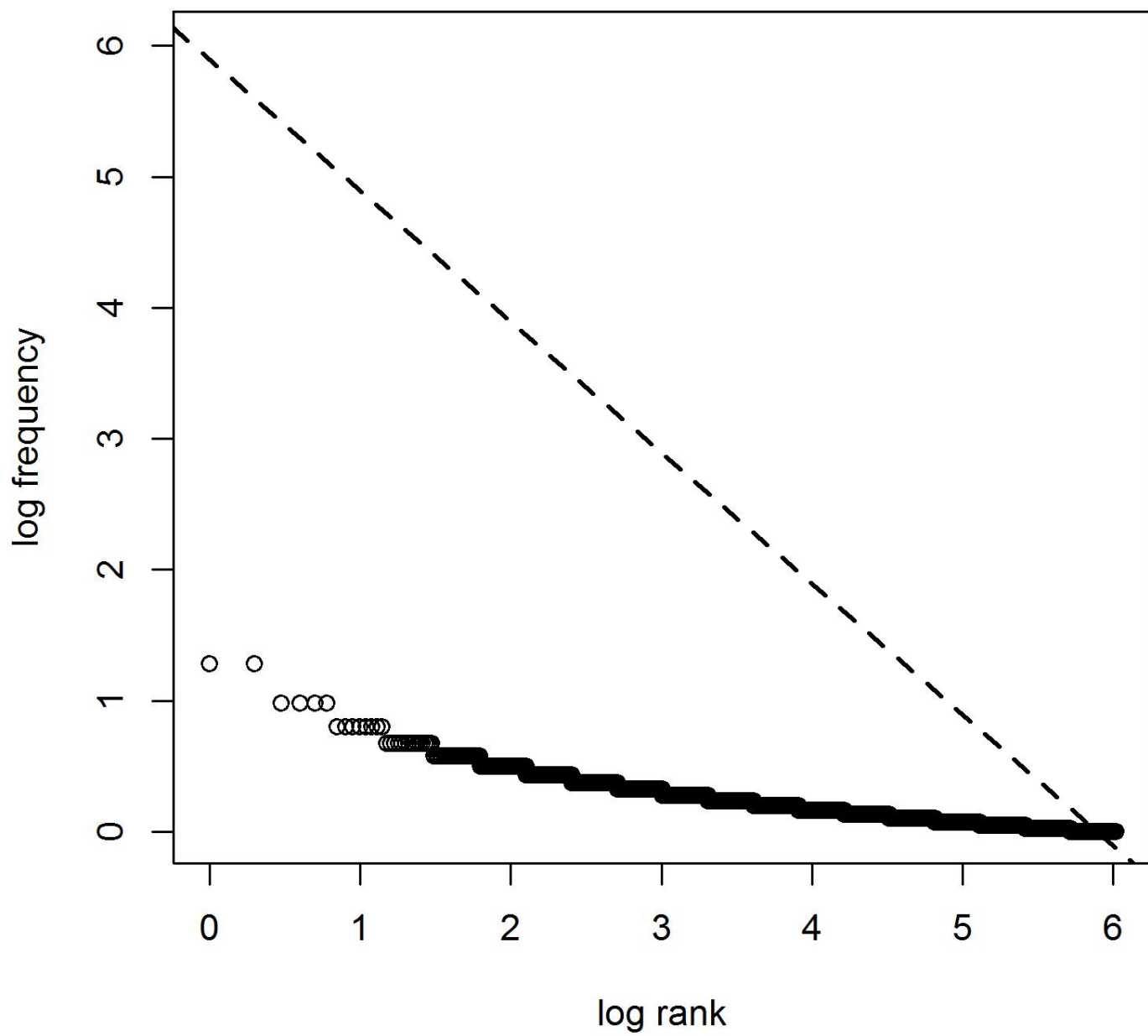

Results for replication of Manin

Supplement: S4 Fig — (PDF) [file pone.0181987.s009.pdf]
